# A direct comparison of next generation sequencing enrichment methods using an aortopathy gene panel- clinical diagnostics perspective

**DOI:** 10.1186/1755-8794-5-50

**Published:** 2012-11-14

**Authors:** Whitney L Wooderchak-Donahue, Brendan O’Fallon, Larissa V Furtado, Jacob D Durtschi, Parker Plant, Perry G Ridge, Alan F Rope, Angela T Yetman, Pinar Bayrak-Toydemir

**Affiliations:** 1 ARUP Institute for Clinical and Experimental Pathology, Salt Lake City, USA; 2 Department of Pathology, University of Utah, Salt Lake City, USA; 3 Department of Pediatrics, Division of Medical Genetics, University of Utah, Salt Lake City, USA; 4 Department of Pediatrics, Division of Cardiology, University of Utah, Salt Lake City, USA; 5 Molecular Genetics Department, ARUP Institute for Clinical and Experimental Pathology, 500 Chipeta Way, Salt Lake City, UT, 84108, USA

**Keywords:** Aortopathy, Hybridization capture, Marfan syndrome, Next generation sequencing (NGS), Target enrichment, Emulsion PCR

## Abstract

**Background:**

Aortopathies are a group of disorders characterized by aneurysms, dilation, and tortuosity of the aorta. Because of the phenotypic overlap and genetic heterogeneity of diseases featuring aortopathy, molecular testing is often required for timely and correct diagnosis of affected individuals. In this setting next generation sequencing (NGS) offers several advantages over traditional molecular techniques.

**Methods:**

The purpose of our study was to compare NGS enrichment methods for a clinical assay targeting the nine genes known to be associated with aortopathy. RainDance emulsion PCR and SureSelect RNA-bait hybridization capture enrichment methods were directly compared by enriching DNA from eight samples. Enriched samples were barcoded, pooled, and sequenced on the Illumina HiSeq2000 platform. Depth of coverage, consistency of coverage across samples, and the overlap of variants identified were assessed. This data was also compared to whole-exome sequencing data from ten individuals.

**Results:**

Read depth was greater and less variable among samples that had been enriched using the RNA-bait hybridization capture enrichment method. In addition, samples enriched by hybridization capture had fewer exons with mean coverage less than 10, reducing the need for followup Sanger sequencing. Variants sets produced were 77% concordant, with both techniques yielding similar numbers of discordant variants.

**Conclusions:**

When comparing the design flexibility, performance, and cost of the targeted enrichment methods to whole-exome sequencing, the RNA-bait hybridization capture enrichment gene panel offers the better solution for interrogating the aortopathy genes in a clinical laboratory setting.

## Background

Aortopathies are a group of disorders characterized by aneurysms, dilation, and tortuosity of the aorta. Thoracic aortic aneurysm with dissection is the most common fatal condition involving the aorta [1], and can be syndromic, familial nonsyndromic, or sporadic. Over 20% of thoracic aneurysms result from inherited disorders [2]. Syndromic connective tissue diseases with aortic involvment, such as Marfan syndrome (MFS; OMIM# 154700), Loeys-Dietz syndrome (LDS; OMIM# 609192), Ehlers Danlos syndrome type IV (EDS IV; OMIM# 130050), and congenital contractural arachnodactyly (OMIM# 121050) each result from mutations in different genes yet have broadly overlapping phenotypes [3-6]. In addition, mutations in genes related to the structure and function of the aortic wall, including *MYH11*[7,8]*, ACTA2*[9]*, SLC2A10*[10]*,* and *NOTCH1*[11], have been linked to non-syndromic familial forms of thoracic aortic aneurysms (reviewed in [12]).

Due to the overlapping phenotypes presented by these disorders genetic sequencing is often required for accurate diagnosis and appropriate clinical intervention. However, comprehensive sequencing of the many genes involved is often impractical with traditional Sanger methods. In contrast, high-throughput “next-generation” sequencing (NGS) has emerged as a new tool in the clinical laboratory for the rapid, cost-effective detection of mutations in genes associated with multigenic disorders [13,14]. NGS assays targeted to a panel of genomic regions associated with known pathogenic mutations offer several advantages over traditional sequencing methods, including lower cost and rapid assessment of many regions.

While NGS offers a promising alternative to Sanger sequencing, the clinical utility of NGS methods depends on the ability to accurately isolate or amplify the genomic regions of interest. Ideally, the enrichment will yield high read depths in the targeted regions while keeping errors introduced through PCR and other sample manipulations to a minimum. Several NGS target enrichment strategies are currently available, all with various advantages depending on the size of the targeted region and the genetic targets themselves. Previous comparisons of enrichment techniques have identified considerable differences in the depth of coverage of targeted regions as well as the number of variants identified [15-17].

In this work we compare two competing enrichment protocols, an emulsion-PCR based technique from RainDance technologies [18] and a method utilizing solution-based RNA-bait hybrid capture marketed as the SureSelect Target Enrichment Platform [19]. Using an aortopathy panel of nine genes (Table 1), we assess the clinical utility of each enrichment technique. We quantify the coverage of the targeted regions and compare the accuracy and overlap of the variants identified. To determine which molecular approach is best suited for routine clinical diagnostics for this disorder, we also compare read depths for the aortopathy genes generated from the two custom-designed gene panel enrichments to those obtained from whole-exome sequencing.

**Table 1:** Aortopathy panel genes

**Gene name**	**Chromosomal locus**	**Protein**	**Disease**	**No. reported mutations**	**Transcript size**	**Exons**
*FBN1*	15q21.1	Fibrillin	Marfan syndrome	601	8616 bp	66
*TGFΒR1*	9q33-q34	Transforming growth factor, beta receptor 1	Loeys-Dietz syndrome; Marfan-like syndrome	28^*a*^	1512 bp	9
*TGFΒR2*	3p22	Transforming growth factor, beta receptor 2	Loeys-Dietz syndrome; Marfan-like syndrome	105^*a*^	1779 bp	7
*COL3A1*	2q31	Collagen type III alpha 1	Ehlers Danlos Type IV	227	4401 bp	51
*MYH11*	16p13.13-p13.12	Myosin heavy chain 11	TAAD^*b*^-patent ductus arteriosus	3	5919 bp	41
*ACTA2*	10q22-q24	smooth muscle actin, alpha 2	TAAD4 syndrome	19	1134 bp	9
*SLC2A10*	20q13.1	solute carrier family 2 (facilitated glucose transporter), member 10	Arterial Tortuosity syndrome	18	1626 bp	5
*NOTCH1*	9q34.3	Notch homolog 1, translocation-associated	BAV-TAAD syndrome	11	7668 bp	34
*FBN2*	5q23-q31	Fibrillin 2	BAV-TAAD syndrome	45	8739 bp	71

^*a*^Of the 28 patients with a *TGFΒR1* mutation, 14 had Loeys-Dietz syndrome, 4 had thoracic aortic aneurysms, and 4 had Marfan-like syndrome. Of the 105 TGFΒR2 mutations, 32 had Loeys-Dietz syndrome, 31 had Marfan-like syndrome, and 8 had thoracic aortic aneurysms.

^*b*^Thoracic aortic aneurysms and dissections (TAAD); Bicuspid-Aortic Valve (BAV).

## Methods

### Samples

Eight samples were used to directly compare the enrichment technologies. Four samples were positive controls with a known mutation in one of the aortopathy genes (Table 2, Samples 1–4). Samples 5 and 6 were from patients with a clinical diagnosis of aortopathy and a negative molecular analysis of *FBN1*, *TGFβR1,* and *TGFβR2* by Sanger sequencing analysis and multiplex ligation-dependent probe amplification (MLPA) [20]. A normal, healthy individual’s DNA in which a mutation in the aortopathy genes was not expected was used as a negative control. A Coriell sample with an *FBN1* c.1888delAAinsC, p.M717X genotype was also evaluated.

**Table 2:** NGS accuracy sample results

**#**	**Sample**	**Mutations**	**Coverage RD^*a*^ (% mutant allele)**	**Coverage SS^*a*^ (% mutant allele)**	**Mean coverage**	**Other information**
1	*FBN1* Positive	c.1585C>T, p.R529X	101x (47%)	576x (60%)	1477x (RD) 508x (SS)	In *COL3A1*, c.198A>G, p.I66M, a novel change.
2	*TGFBR1* Positive	c.799A>G, p.N267D	317x (51%)	504x (58%)	462x (RD) 563x (SS)	
3	*TGFBR2* Positive	c.1583G>A, p.R528H	183x (48%)	257x (50%)	177x (RD) 499x (SS)	
4	*MYH11* Positive	IVS32+1C>A (splice)	71x (58%)	80x (50%)	140x (RD) 164x (SS)	
5	Symptomatic unknown	No mutations found.	NA	NA	33x (RD) 260x (SS)	In *SLC2A10* p.A206T (rs#2235491) and p.A385G (rs#79849424). In *NOTCH1*, IVS16-4C>CT (rs#3125001).
6	Symptomatic unknown	*COL3A1* IVS9-7T>C novel change; potential splice site variant	48x (56%)	416x (50%)	86x (RD) 355x (SS)	In *COL3A1*, p.A698T (rs#1800255). In *FBN2*, p.V965I (rs#154001).
7	*FBN1* Coriell positive	Unable to confirm due to sample quality.	Failed merge between DNA and primer library droplets	Poor data due to low quality DNA	NA	
8	Healthy Control	No mutations found	NA	Failed due pipetting error	340x (RD)	

^*a*^RainDance (RD) and SureSelect (SS). All variants reported in this table are heterozygous as confirmed by Sanger sequencing (data not shown). NA, not applicable.

This study was approved by the University of Utah and Primary Children’s Medical Center Institutional Review Boards (IRB#00028740). Written informed consent for participation in the study was obtained from the participants or their parents.

### Emulsion PCR enrichment

A primer library (RainDance Technologies, Boston, MA) was custom-designed to amplify 194 exons and exon/intron boundaries for nine aortopathy genes listed in Table 1 (~0.1 Mb). Primers were designed using RainDance’s design parameters and Primer3 (http://primer3.sourceforge.net/). The 350 PCR amplicons ranged in size from 201–919 bp and had a guanine cytosine (GC) content of 24-80%. Genomic DNA (1.5-3 μg) was sheared to 2–4 kb fragments using a Covaris S2 instrument (Covaris, Woburn, MA) and added to a mixture that included all the components of the PCR reaction excluding the primers. This mixture and the primer library were loaded separately onto the RDT1000 instrument, and PCR droplets containing one primer pair per droplet were generated. After amplification, emulsion PCR droplets were broken releasing the amplicons which were then purified and concatenated according to the manufacturer’s instructions. Concatenated samples were sheared to 300 bp fragments, and Illumina adapters were added using the SPRI-TE instrument (Beckman-Coulter, Danvers, MA). Barcode indexes were added using PCR, and sample quality and quantity was assessed using a Bioanalyzer (Agilent Technologies, Santa Clara, CA).

### Solution-based hybridization capture enrichment

Custom RNA baits were designed to specifically target the exons and exon/intron boundaries of the nine aortopathy genes from Table 1 (~0.1 Mb). RNA baits were tiled at 5 × spacing and were in replicates of 10 to increase hybridization efficiency. Genomic DNA (3 μg) was sheared to 180 bp fragments. Illumina adapters were added using SureSelect XT kit reagents (Agilent Technologies, Santa Clara, CA). Adapter ligated DNA underwent hybridization with the biotinylated RNA baits for 24 hours at 65°C. Hybridized DNA targets of interest were captured using streptavidin-coated magnetic beads. DNA targets of interest were eluted and barcode/indexed after a series of washes to remove the non-targeted, unbound genome. DNA quality and quantity was assessed (Bioanalyzer).

### NGS sequencing and data analysis

Concentrations of indexed samples from the enrichments were determined by quantitative-PCR (KAPA Biosystems, Woburn, MA). Samples were pooled and sequenced on a HiSeq2000 instrument (Illumina, San Diego, CA) using 2 × 100 paired-end sequencing. Sequences were aligned to the human genome reference (hg19) sequence using the Burrows-Wheeler Alignment tool (BWA 0.5.9) [21] with default parameters. PCR duplicates were removed using the Samtools package [22], and base quality score recalibration, local realignment, and variant calling were performed with the Genome Analysis Toolkit (GaTK v1.3) [23]. Variants with coverage less than 6, with 'quality-by-depth' scores of less than two, with variant allele frequencies of less than 0.15, or with overall quality scores of less than 10 were discarded.

### Sanger sequencing

Select variants were Sanger sequenced. Primers sequences are available upon request. Amplicons were bi-directly sequenced using the Big Dye® Terminator v3.1 cycle sequencing kit and an ABI 3730 DNA Analyzer (Life Technologies, Carlsbad, CA). Sequences were compared to reference sequences using Mutation Surveyor (SoftGenetics, State College, PA).

### Exome sequencing

In addition to the custom targeted enrichments, we also examined depth of coverage for the targeted regions obtained through exome sequencing. Briefly, ten samples from individuals without known aortopathies were sequenced on the Illumina HiSeq2000 platform after undergoing exome capture enrichment (SureSelect 50Mb exome kit, Agilent Technologies, Santa Clara, CA). Exome samples were indexed and pooled, two per lane, using a one-to-one ratio, and were sequenced using 2x100-base pair paired end reads.

## Results

### Sample enrichment

Six of eight samples underwent successful enrichment using both RainDance emulsion PCR and SureSelect hybridization capture (Table 2). Sample 7 (from Coriell) failed during the RDT1000 merge when the DNA droplets failed to merge properly with the primer library droplets. Hybridization capture of this sample was also poor and analysis was not completed due to sub-optimal DNA quality (A_260_/A_280_ was not between 1.8 and 2.0). Sample 8 failed during hybridization capture due to a pipetting error. Herein, the accuracy of the two enrichment methods, depth of coverage, and variant calling were compared among the six samples enriched using both techniques.

### Accuracy

Pathogenic mutations from four positive control samples were detected using both enrichment methods (Table 2, samples 1–4). For example, a heterozygous *FBN1* c.1585C > T, p.R529X mutation was detected in sample 1 using both enrichment methods and was confirmed using Sanger sequencing (Additional file 1: Figure S1).

### Depth of coverage

**Figure 1:**
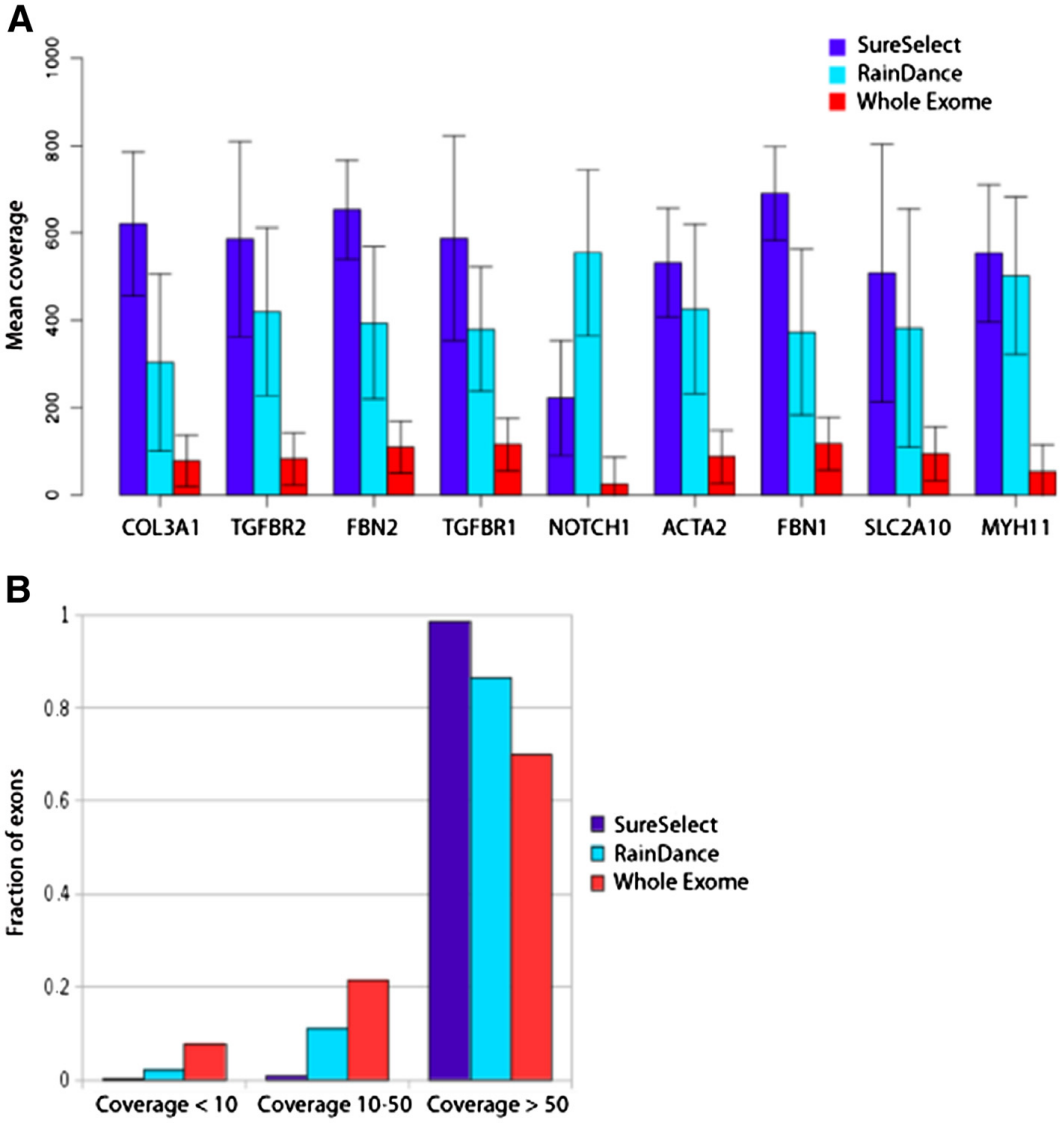
**Mean depth of coverage per aortopathies gene for the enrichment methods.** In **A**, both custom-designed gene panels (SureSelect and RainDance) yielded significantly higher average coverage per gene compared to whole-exome sequencing (SureSelect 50 Mb All Exon Capture). Bars indicate one standard deviation. In **B**, the fraction of exons covered to the given depth for the enrichment methods is shown. The custom-designed SureSelect gene panel yielded greater average read depths across the 294 exons for all samples versus the RainDance or whole-exome enrichments.

Omitting failed samples, both methods generated very high coverage in the targeted regions. Pooling data from all samples and examining coverage on a gene-by-gene basis, the SureSelect enriched material yielded slightly higher, but also more variable, coverage (per-gene average 471.7, range 189.9-592.0) compared to the emulsion PCR technique (per-gene average 414.8, range 303.4-554.4). Both methods yielded significantly higher average coverage per gene than whole-exome sequencing (Figure 1A) and had substantially fewer exons with less than tenfold coverage (Figure 1B). SureSelect results were influenced by particularly low coverage for the *NOTCH1* gene (mean 189x), a feature not observed with the sequences generated by emulsion PCR. Low coverage may be related to the GC content of the *NOTCH1* gene, in which 17 of 34 exons are > 65% GC.

Examining exons individuals, the SureSelect enrichment yielded fewer total exons with coverage below 10 when compared to both RainDance and the whole exome capture (Figure 1B). Some 98.5% of all exons had mean depth greater than 50 for SureSelect, as compared to 85.5% for RainDance and 70% for whole exome.

**Figure 2:**
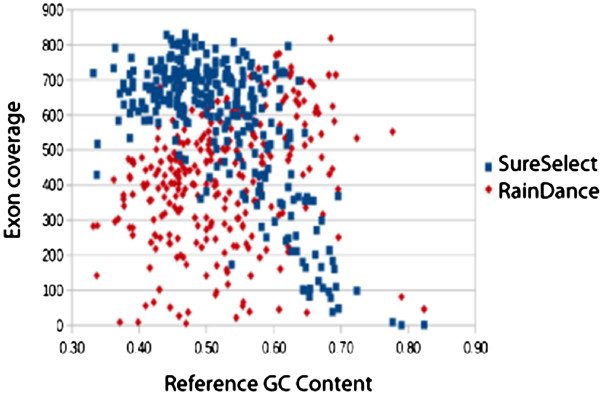
**Mean exon read depth as a function of reference sequence GC content for the custom-designed gene panels.** High GC content resulted in decreased exon coverage in the SureSelect enriched samples. GC content had little to no effect on exon coverage of the RainDance enriched samples, which were more prone to sporadic amplicon failure.

Despite relatively high read depths across most exons, several exons consistently had little or no coverage among the samples. Within the SureSelect-enchriched sequences, much of the variation appears to be related to the GC content of the surrounding sequence (Figure 2). For exons containing greater than 75% GC (for instance, exon 1 in the *NOTCH1* and *TGFβR1* genes) no coverage was observed.

**Figure 3:**
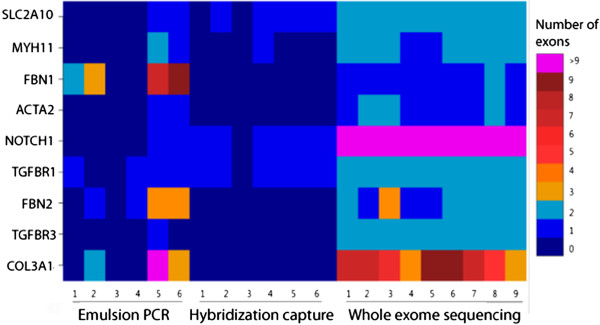
**Number of exons with mean read depth less than 20, across samples (x-axis) and genes (y-axis).** The color of the block represents the number of exons with low coverage for a given sample and gene.

**Figure 4:**
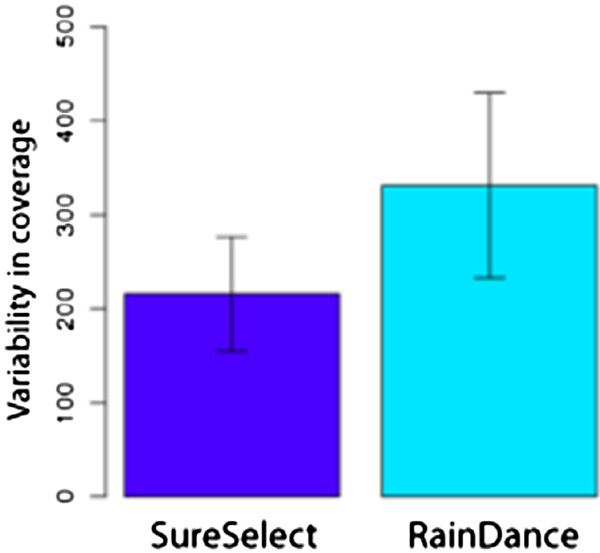
**Variability in coverage: RainDance versus SureSelect.** Standard deviation in coverage for each exon across enrichment samples was computed. The mean deviation across exons reflects the overall degree of coverage variability across samples 1–6, with zero indicating that each exon was covered by the same number of reads in every sample. Between sample variability was significantly higher for the RainDance enrichment.

In contrast to the strong correlation of GC content with read depth for the hybridization capture enrichment, exon coverage varied in an unpredictable manner for the emulsion PCR enrichment method (Figure 3). To assess the degree of coverage variability across samples, we computed the standard deviation in coverage for each exon across samples for both custom-designed panel enrichment techniques. The mean of these deviations across exons reflects the overall degree of coverage variability across all samples, with zero indicating that each exon was covered by the same number of reads in every sample. Between sample variability was significantly higher for the emulsion PCR enrichment, with a mean of 330.4, compared to 215.8 for the hybridization enrichment panel (paired *t*-test, p < 2.2 × 10^-16^, Figure 4).

### Variants analysis

In addition to the number of reads mapping to targeted locations, we also assessed the quantity and character of the variants identified by both enrichment methods. Because both methods targeted overlapping, but partially distinct regions, we considered only those variants in the intersection of the two capture regions. The intersection of the two capture regions was 60.58 Kb in size, comprising 64% of the bases targeted by SureSelect and 99% of bases in the RainDance capture.

In total, 252 unique variants were identified across all samples, 195 of which were identified by both methods (Additional file 1: Table S1). The sequences generated by the RainDance enrichment contained several more variants, 221 in total, including 26 not identified in the SureSelect-enriched sequences. In contrast, the SureSelect protocol yielded 214 total variants, of which 19 were not found using the emulsion PCR technique. Among the variants common to both methods, 55 (28.2%) were in dbSNP (v. 132), while 2 of the 17 variants (12%) unique to SureSelect were in dbSNP, and 3 of the 23 variants (13%) unique to emulsion PCR were in dbSNP. Assuming variants found in dbSNP are not false positives in our samples, this suggests that even the relatively high coverage obtained by both enrichment methods did not guarantee accurate variant detection, as both the custom targeted enrichment methods identified several variants not recognized by the other method. Similarly discordant results have been found in other recent studies [13].

Examination of the discordant variants suggested several sources for the disagreement. First, discordant variants appeared in the intronic and untranslated areas at the periphery of individual capture regions. Ten SureSelect and fifteen RainDance discordant variants were located in non-exonic regions. Examination of read depth in these areas suggested that coverage was relatively low compared to the mean for the whole exon, implicating variation in read depth as a potential source of discordant or false positive calls.

A second source of discordant variation was the *NOTCH1* gene. A relatively high fraction of the discordant variants appeared in the exons of *NOTCH1* (15 of 45, or 33%), and these appeared with similar frequencies in both the RainDance and SureSelect samples (8 from RainDance, 7 from SureSelect). *NOTCH1* is likely to be problematic for two reasons. First, the high GC content of *NOTCH1* reduces the read depths obtained with the SureSelect enrichment procedure. Second, *NOTCH1* bears substantial sequence homology to other *NOTCH* genes, potentially confounding alignment algorithms and generating spurious variant calls.

### Clinical utility of the aortopathy panel

Two clinically affected individuals who had previously tested negative for a *FBN1*, *TGFBR1*, and *TGFBR2* mutation by Sanger sequencing and MLPA [20] were evaluated using the aortopathy panel. Sample 5 was from a 19-year-old female with borderline aortic dilatation, mild tricuspid valve prolapse and mild mitral valve insufficiency, height of 178 cm (5’10”) (99^th^ percentile), arm span of 176 cm (5’9”) severe pectus carinatum, scoliosis, arachnodactyly, joint mobility, positive wrist and thumb signs, and skin striae. Analysis of the coding region variants and splice site variants from both enrichment methods revealed two nonsynonymous heterozygous variants in *SLC2A10* (c.616G > A, p.Ala206Thr and c.1154C > G, p. Ala385Gly) and a *NOTCH1* potential splice site variant (IVS16-4C > T). These variants were present in dbSNP and are likely not disease causing.

Sample 6 was from a 34-year-old female with mild aortic dilatation in the setting of past aortic dissection and unknown family history. Two heterozygous nonsynonymous variants, one in *COL3A1* (c.2092G > A, p.Ala698Thr) and the other in *FBN2* (c.2893G > A, p.Val965Ile) were both present in dbSNP, rs1800255 and rs154001 at 19.8% and 72.4% frequencies in 1000 genomes (November 2010 release, 1000genomes.org), respectively. A novel heterozygous *COL3A1* variant (IVS9-7T > C) not found in dbSNP or the *COL3A1* locus specific mutation database was also identified. Splice site prediction programs were run to determine if the change altered splicing [24], but they predicted that no change in splicing would occur. It is likely that this variant is also not disease causing.

No synonymous splice site variants were identified in either patient. It is possible that these two patients have a large deletion or duplication of one of the nine aortopathy genes, but this has yet to be evaluated. Currently, detection of structural variation cannot be reliably detected from NGS data alone. Patient samples that test negative from the NGS assay should be run on an exonic level comparative genomic hybridization (CGH) array assay used to evaluate large copy number changes in the same gene set. Until NGS data analysis programs improve in their ability and accuracy to detect structural variation, CGH array assays are the suggested approach for detecting such large deletions or duplications in multi-gene panels. Alternatively, another gene not included in the aortopathy panel may be responsible for the disorder in these patients.

## Discussion

### Enrichment performance

All targeted enrichment methods evaluated yielded very high read depths in the aortopathies targeted regions, with all genes except for *NOTCH1* from the exome capture obtaining a mean depth of coverage of over 50 × (Figure 1). Overall, the custom-designed hybridization capture enrichment yielded lower coverage in regions of high GC content (Figure 2), but consistently high coverage in regions of low GC bias. The emulsion PCR method, in contrast, yielded exons with very low coverage that had no obvious correlate (Figure 2). Although influenced by GC content, the hybridization capture nonetheless yielded fewer total exons with low coverage than did the emulsion PCR technique (Figure 1B). Overall, emulsion PCR yielded significantly higher between-sample variability in coverage. In a clinical setting, a high degree of between sample consistency is desirable. Low coverage exons will likely require follow-up Sanger sequencing. Knowing which exons will require such treatment beforehand may ameliorate the cost and additional time resulting from designing new primer sets on an as-needed basis.

Despite satisfactory coverage, the methods yielded only partially overlapping sets of variants, with some 10% of variants identified by one method but not by the other (Additional file 1: Table S1). These inconsistencies may be related to variability in read depth as well as alignment ambiguity related to sequence homology. Similarly high discordancy rates were found in a recent study comparing the RainDance and Fluidigm enrichment platforms [13], in which only 42% of variants identified were common to both methods, and 67% of the discordant variants were found in regions of less than 20-fold coverage. Until procedures are devised that substantially decrease the number of regions with low coverage, Sanger confirmation of suspected variants will likely be necessary prior to clinical action.

A high sample failure rate of 25% (2 of 8 samples) was observed due to sample quality (sample 7, RainDance and SureSelect) and a pipetting error (sample 8, SureSelect) (Table 2). Based on our results, only DNA samples of high quality (A_260/280_ is between 1.8 and 2.0) should be evaluated using NGS enrichment. If a sample falls below this initial quality measurement, DNA should be extracted again and/or the sample should be purified until an acceptable quality is obtained. For complex enrichment protocols such as the SureSelect method, pipetting errors can be avoided by incorporating automation into sample preparation (discussed further below). This is especially important as the number of clinical samples increase.

### NGS enrichment in the clinical laboratory

When compared to emulsion PCR, the solution-based hybridization technique yielded higher coverage as well as greater predictability in performance. These attributes, when combined with lower cost, strongly favor the hybridization capture for enrichment of the aortopathy panel in the clinical setting. Emulsion PCR enrichment is significantly more expensive and requires the use of a costly instrument. Exome sequencing is also more costly because the cost to sequence the exome at a high enough depth of coverage to accurately call variants is much higher than for the targeted panel. Samples can also be indexed and pooled prior to hybridization capture [25], reducing the overall cost per sample even further.

Despite the lower cost, the hybridization capture protocol is more time consuming, complex, and labor intensive when compared to the emulsion PCR technique. However, the ability to automate this process in the clinical laboratory alleviates these challenges and yields more consistent, reliable results [26]. Currently, the emulsion PCR instrument processes one sample/hour, limiting the total number of samples processed in a day to approximately twenty-four. With the increasing interest in exome sequencing, automation of hybridization capture enrichment protocols have become readily available [26], and up to 96 different custom-designed NGS panels and exome hybridizations can take place at the same time during the same run. Solution-based hybridization libraries can be ordered in larger volumes, aliquoted for up to two-three freeze/thaw cycles, and stored for up to one year. RainDance primer libraries have to be switched out after the eighth sample with the RDT1000 instrument design, and if there are not eight samples to run using the same primer library, the rest will be wasted.

## Conclusions

The aortopathy panel offers a cost-effective, faster molecular diagnostic assay compared to the conventional gene-by-gene Sanger sequencing approach. Both RainDance and SureSelect enrichment methods accurately identified variants in the positive samples assayed, and overall coverage was high across most exons for all samples. Hybrid capture results demonstrated that genes with high homology to other genes and high GC rich regions can be problematic. However, we note that increases in read length, currently available with the Ion Torrent or Illumina MiSeq instruments, are likely to alleviate homology-related alignment issues. After comparing the cost, design flexibility, and versatility of the workflows of the two enrichment methods, the custom-designed hybridization capture design offers the best solution for enriching the aortopathy genes in a clinical laboratory setting. Patients with Marfan syndrome and Marfan-like syndromes featuring aortopathies will benefit from the timely molecular diagnosis of this new testing approach which will lead to the appropriate surveillance and interventions aimed at preventing the significant morbidity and mortality associated with these conditions.

## Competing interests

We have no financial, nonfinancial, or competing interests to declare.

## Authors' contributions

WLWD participated in the design of the study, performed all experiments, assisted in data analysis, and wrote the manuscript; BO analyzed the data and wrote the manuscript; LVF participated in the design of the study and wrote the manuscript; JDD and PGR assisted in data analysis; PP assisted with experiments and data analysis; AFR and ATY were responsible for diagnosis and management of patients and participated in the design of the study; PBT conceived the study, and participated in its design and coordination. All authors read and approved the final manuscript.

## Pre-publication history

The pre-publication history for this paper can be accessed here:

http://www.biomedcentral.com/1755-8794/5/50/prepub

## Supplementary Material

Additional file 1 **Figure S1. **NGS and Sanger sequencing confirm the pathogenic *FBN1* mutation in sample 1. A heterozygous nonsense mutation (c.1585C>T, p.R529X) was detected using RainDance PCR enrichment (panel A) and SureSelect capture enrichment (panel B). In C, Sanger sequencing confirmed the mutation detected in both enrichment strategies. The *FBN1* gene is on the reverse strand, and appears in the 3’ to 5’ orientation in the NGS traces versus the Sanger sequencing trace which is 5’ to 3’. **Table S1**. Summary of variants detected from the RainDance and SureSelect enrichments. Click here for file
