# Supplementary material for: A direct comparison of next generation sequencing enrichment methods using an aortopathy gene panel- clinical diagnostics perspective
Source: BMC Med Genomics. 2012 Nov 14;5:50. doi: 10.1186/1755-8794-5-50 (PMC3534588; doi:10.1186/1755-8794-5-50)
Supplement: Additional file 1 — Figure S1. NGS and Sanger sequencing confirm the pathogenic FBN1 mutation in sample 1. A heterozygous nonsense mutation (c.1585C>T, p.R529X) was detected using RainDance PCR enrichment (panel A) and SureSelect capture enrichment (panel B). In C, Sanger sequencing confirmed the mutation detected in both enrichment strategies. The FBN1 gene is on the reverse strand, and appears in the 3’ to 5’ orientation in the NGS traces versus the Sanger sequencing trace which is 5’ to 3’. Table S1. Summary of variants detected from the RainDance and SureSelect enrichments. [file 1755-8794-5-50-S1.docx]

**Supplementary Data**

**Figure 1.**

**
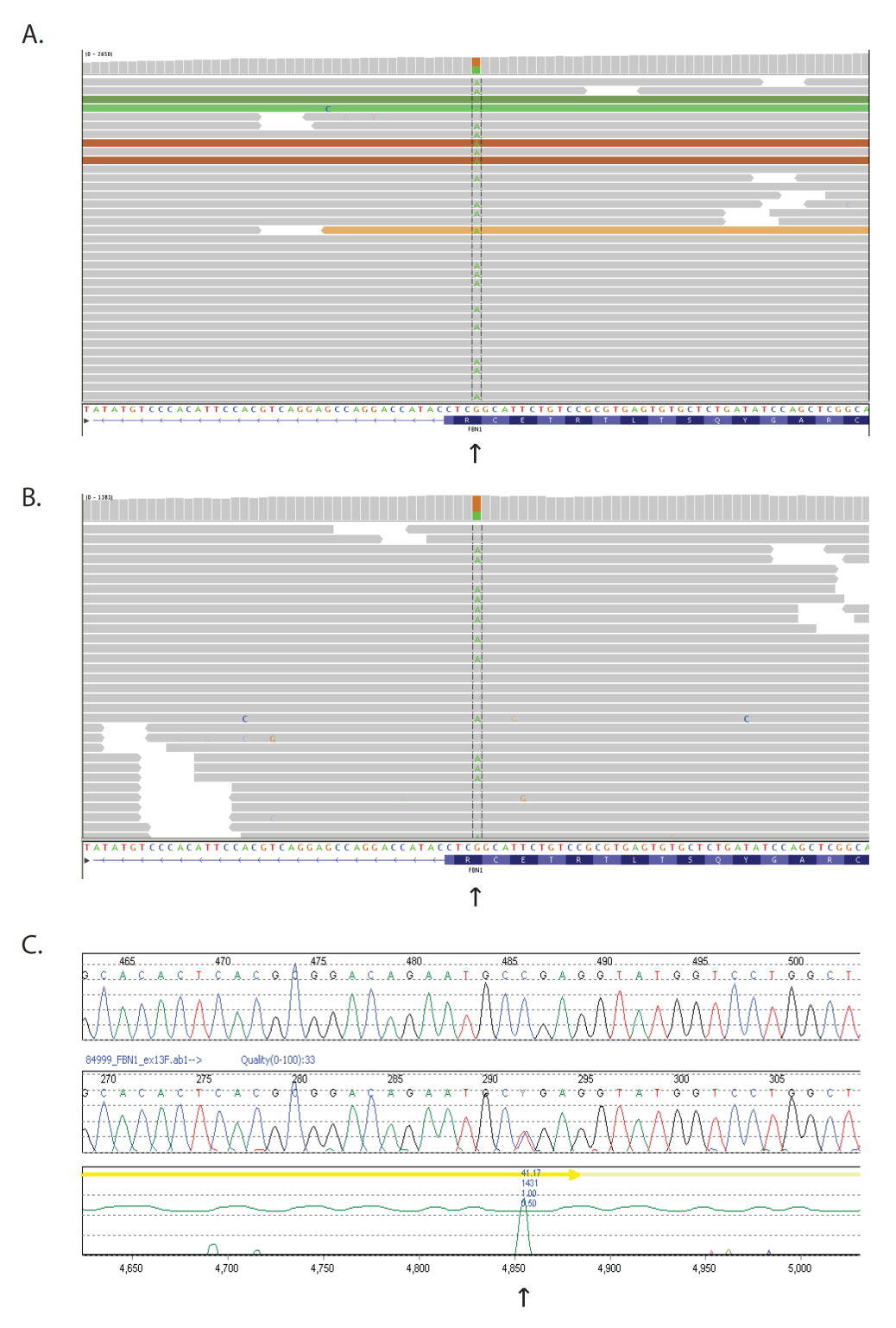
**

**Figure 1. NGS and Sanger sequencing confirm the pathogenic *FBN1* mutation in sample 1.** A heterozygous nonsense mutation (c.1585C>T, p.R529X) was detected using RainDance PCR enrichment (panel A) and SureSelect capture enrichment (panel B). In C, Sanger sequencing confirmed the mutation detected in both enrichment strategies. The *FBN1* gene is on the reverse strand, and appears in the 3’ to 5’ orientation in the NGS traces versus the Sanger sequencing trace which is 5’ to 3’.

**Table 1. Summary of variants detected from the RainDance and SureSelect enrichments.**

| **Sample** | **Unique -SureSelect** | | **Both Methods** | **Unique- RainDance** | | |
| --- | --- | --- | --- | --- | --- | --- |
|  | **dbSNP** | **Novel** |  | **dbSNP** | **Novel** | |
| 1 | 0 | 1 | 33 | 2 | | 9 |
| 2 | 0 | 0 | 30 | 0 | | 3 |
| 3 | 0 | 1 | 28 | 0 | | 4 |
| 4 | 0 | 4 | 34 | 1 | | 3 |
| 5 | 0 | 4 | 38 | 0 | | 0 |
| 6 | 2 | 7 | 32 | 0 | | 4 |
| **Total** | **2** | **17** | **195** | **3** | | **23** |
